# Comparison of self-reported & device-based, measured physical activity among children in Germany

**DOI:** 10.1186/s12889-021-11114-y

**Published:** 2021-06-05

**Authors:** Alexander Burchartz, Doris Oriwol, Simon Kolb, Steffen C. E. Schmidt, Kathrin Wunsch, Kristin Manz, Claudia Niessner, Alexander Woll

**Affiliations:** 1 Institute of Sports and Sports Science, Karlsruhe Institute of Technology, Engler-Bunte-Ring 15, Building 40.40, 76131 Karlsruhe, Germany; 2 Robert Koch Institute, Nordufer 20, 13353 Berlin, Germany

**Keywords:** *Accelerometer*, *Cross table heat maps*, *MoMo*, *KiGGS*, Adolescents, Guideline

## Abstract

**Background:**

As children show a more complex but less structured movement behavior than adults, assessment of their many spontaneous and impulsive movements is a challenge for physical activity (PA) assessment. Since neither questionnaires nor accelerometers enable optimal detection of all facets of PA, a multimodal, combined approach of self-reported and device-based methods is recommended. Based on the number of days on which the participants reached the physical activity (PA) values given in the WHO guideline, this study examines 1) the difference between self-reported and device-based, measured PA and 2) whether PA differences between age and gender groups obtained by two methods are comparable.

**Methods:**

Participants aged 6–17 years were randomly chosen and data were collected representatively at 167 sample points throughout Germany within the Motorik-Modul Study. PA of *n* = 2694 participants (52.3% female) was measured using the ActiGraph accelerometer (ACC) and a physical activity questionnaire (PAQ). The sample was divided into three age groups (6–10 yrs. *n* = 788, 11–13 yrs. *n* = 823, 14–17 yrs. *n* = 1083). Numbers of days per week with at least 60 min moderate to vigorous PA (MVPA) were analyzed for both methods.

**Results:**

Only every 25th respondent (4%) reaches the WHO standard of 60 min MVPA every day if measured with ACC. Self-reported PA was slightly higher (9%) (mean_PAQ_ = 3.82 days; mean_ACC_ = 2.34 days; *F*_*method*_ = 915.85; *p =* <.001; *f*_*Cohen*_ = .64). The differences between the methods are significantly smaller in younger children than in the older age groups (*F*_*age*_ = 264.2, *p* < .001; *f*_*Cohen*_ = .48). The older the subjects are, the lower is the proportion of those who meet the WHO guideline on each day, with girls meeting the guideline less frequently than boys in all age groups.

**Conclusion:**

Children and adolescents living in Germany show a very low adherence to the WHO guideline on PA. While younger children are much more active with their free play, especially children over 10 years of age and especially girls should be the target of programs to increase PA.

**Supplementary Information:**

The online version contains supplementary material available at 10.1186/s12889-021-11114-y.

## Background

As children show a more complex but less structured movement behavior than adults [1, 2], assessment of their many spontaneous and impulsive movements is a challenge for physical activity (PA) assessment [3]. To date, questionnaires have been the most commonly used method to assess PA in large, epidemiological studies. One of the biggest advantages of questionnaires lies in their versatility. In addition to the assessment of duration, frequency, and intensity, self-report methods provide information about the type of PA, which is not feasible with common device-based methods like accelerometry without complex diaries or ambulatory assessment. Furthermore, epidemiological research requires larger samples, which is why questionnaires often are the more feasible alternative [4]. However, many studies have shown that the level of PA assessed by self-reports is often overestimated [4–6]. Since this overestimation is higher in children and adolescents compared to adults, it is particularly important to make accurate investigations in the former group [5]. Unstructured and irregular activities in everyday life are difficult to remember correctly. Accelerometers were used more frequently to measure PA in recent large-scale studies [7], as they became more feasible, more accurate, and more affordable in the last decade. By measuring movement acceleration, everyday PA, including PA intensities and patterns, can be recorded in more detail than by self-reports.

In conclusion, no single procedure provides for optimal detection of all facets of PA, which is why a multimodal, combined approach of self-reported and device-based methods is recommended [8, 9].

To the best of our knowledge, this study is the first attempt to compare self-reported and device-based, measured PA guideline adherence in a nationwide sample of children and adolescents living in Germany. Based on the number of days on which the participants reached the value given in the World Health Organization (WHO) guideline [10], this study examines 1) the difference between self-reported and device-based, measured PA, and 2) whether PA differences between age and gender groups are comparable in the two methods.

## Methods

### Study design

The German Health Interview and Examination Survey for Children and Adolescents (KiGGS) is part of the Federal Health Monitoring System conducted by the Robert Koch-Institute (RKI) and consists of regularly conducted nationwide surveys among children, adolescents, and young adults aged 0 to 29 years and living in Germany. KiGGS Wave 2 was conducted between 2014 and 2017. The Motorik-Modul Study (MoMo) is a submodule of the KiGGS study and aims to assess physical fitness, PA, as well as determinants of PA in children and adolescents [11].

The whole study sample was drawn from the German resident population aged 4 to 17 years using a two-stage cluster sampling approach. Informed consent to participate in the study was obtained from all parents of the participants. Also, participants from the baseline study (2003–2006) and Wave 1 (2009–2012) were reinvited. A detailed description of the study design and sampling procedure can be found elsewhere [11–13]. KiGGS and MoMo provide nationally representative data of PA and sedentary behavior of children, adolescents, and young adults living in Germany. A positive vote of the ethics committee of Karlsruhe Institute of Technology of September 23, 2014, is available for the study.

### Sample description

For the current analysis, only cross-sectional data of participants aged 6 to 17 years from KiGGS and MoMo Wave 2 (2014–2017) were used (*n* = 2743). A detailed dropout description can be found elsewhere [14]. To investigate the direct comparison of both measurement methods within each participant, only participants with complete valid device-based (using accelerometer) data as well as self-reported (via physical activity questionnaire) PA data were included in the analyses. The final sample consisted of *n* = 2236 children and adolescents (mean_age_ = 12.5, SD = 3.3; Table 1). The sample was divided into three age groups (6–10 yrs. *n* = 698, 11–13 yrs. *n* = 694, 14–17 yrs. *n* = 844) as well as two gender groups (boys *n* = 1050, girls *n* = 1186). The sample revealed no gender differences in age, weight, height, or BMI.

**Table 1:** Characteristics of participants: Number of participants as n(%). Age in years, weight in kg, height in cm, body mass index in kg /m^2^, data are presented as mean ± SD

Variable	Overall									6–10 years old						11–13 years old						14–17 years old					
	All			Boys			Girls			Boys			Girls			Boys			Girls			Boys			Girls		
n	2236	(100)		1050	(47)		1186	(53)		350	(15.7)		348	(15.6)		321	(14.4)		373	(16.7)		379	(16.9)		465	(20.8)	
Age, yr	12.5	±	3.3	12.4	±	3.3	12.7	±	3.3	8.5	±	1.4	8.5	±	1.4	12.5	±	0.9	12.5	±	0.8	15.9	±	1.1	16.0	±	1.1
Weight, kg	48.4	±	17.5	48.4	±	19.0	46.7	±	15.7	30.1	±	7.3	30.1	±	8.3	48.3	±	11.8	47.2	±	10.6	67.1	±	13.7	59.4	±	11.0
Height, cm	154.2	±	18.0	155.5	±	20.0	152.9	±	16.0	133.7	±	9.6	133.2	±	10.9	157.4	±	9.9	156.6	±	8.6	176.0	±	8.9	165.5	±	6.3
BMI, kg·m^−2^	19.3	±	3.8	19.2	±	3.8	19.3	±	3.8	16.7	±	2.4	16.7	±	2.7	19.3	±	3.3	19.1	±	3.2	21.6	±	3.8	21.7	±	3.5

### Measures

#### Self-reported PA data – physical activity questionnaire (PAQ)

The MoMo-PAQ is a self-administered questionnaire with 28 items [15]. The reliability and validity of the MoMo-PAQ were found to be comparable to those of other activity questionnaires internationally used to assess PA for the same age group. Data obtained with the MoMo-PAQ are sufficiently reliable (test-retest reliability: *ICC* = 0.68), but correlation coefficients with accelerometry data are low (*r* = 0.29) [4].

The goal of the MoMo-PAQ is a domain-specific quantification of PA in minutes per week at different intensity levels (low, moderate, and vigorous) in four domains: PA in everyday life, PA in school, leisure time in sports clubs, and leisure time outside of sports clubs. For the present study, the guideline adherence question “On how many days of a normal week were you/was your child physically active for at least 60 minutes” was analyzed [16]. Parents (or legal guardians) of the 6- to 10-year-olds helped to complete the self-administered questionnaires, the 11- to 17-year-olds did so themselves. The answer categories ranged from 0 to 7 days. This question refers to the internationally agreed PA criterion of at least 60 min of moderate to vigorous PA per day that is promoted by the World Health Organization (WHO) [10, 17].

#### Device-based, measured PA data - accelerometer

For assessment of device-based, measured PA, ActiGraph GT3X accelerometers were used, as they were found to be reliable and valid devices to monitor PA in children and adolescents [18–22]. The technical and methodological details of the present study, which are required when accelerometers are used in epidemiological studies (suggested by [23]), can be found elsewhere [14]. A modified summary of the [14] setup procedures can be found in Table 2. For the present study, minutes of moderate to vigorous-intensity physical activity (MVPA) per day were calculated. Each day was categorized as either meeting the guideline (MVPA > 60 min.) or not meeting the guideline. The resulting variable, therefore, ranges from 0 to 7.

**Table 2:** Expanded list of accelerometer criteria used in KiGGS and MoMo, modified from [14]

Criteria	Definitions within this study
Accelerometer devices	ActiGraph (models: GT3X+, wGT3X-BT)
Placement of the device	Laterally on top of the right anterior superior iliac spine
Sampling frequency	30 Hz
Filter	Normal ActiGraph GT3X filter
Epoch lengths	1 s with possibility to convert into 5 s, 10s, 15 s, 30s and 60s
Nonwear time definition	[24]: 90-min time window for consecutive zero/nonzero counts; allowance of 2-min interval of nonzero counts with a up/downstream 30-min consecutive zero counts window
Valid days/valid weeks	8 h of recordings on four weekdays and one further weekend day when wearing the device for 7 days
Population age range	Children, adolescents and young adults from 6 to 17 years
Sedentary and physical activity intensity classification and cut point algorithms	6–10 years: [25] 11–17 years: [26]

### Statistical analysis

**Fig. 1:**
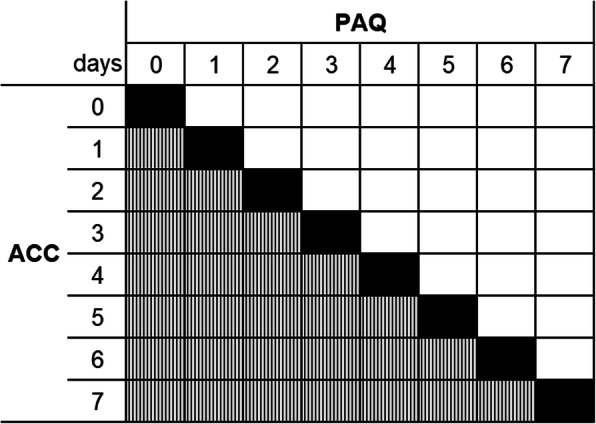
Cross table heat map - black cells: Same results in the PAQ and ACC; white cells: More days with the PAQ; vertically lined cells: More days with the ACC

Statistical analyses were performed using SPSS Statistics 24 (IBM Corporation, Armonk, NY). Differences in the number of days with 60 min of MVPA (dependent variable) between the assessment methods (independent variables) were calculated. Repeated measures ANOVAs were calculated for assessment methods (PAQ and ACC) with between-subjects factors age and gender to reveal the effects of assessment and interaction. Mean values, standard deviations, as well as *p*-values, and effect sizes (f) are given for the analysis of variance. 0.1 stands for a small effect, 0.25 for a medium, and 0.4 for a large effect [27]. The statistical significance level was set to .05. 95% CI were calculated for the comparison of all age and gender groups. Crosstable heat maps were chosen to display two-dimensionally varying PAQ and ACC values of WHO guideline adherence by the same subject. Those heat maps are color-coded (white-gray-black for low to high values) for each cell to visually highlight patterns in row-column interactions. Figure 1 symbolizes compliance with the standards as shown by the PAQ and the ACC. When a participant met the criteria outlined in the PAQ, he/she can be found on the black-colored diagonal. When the participant reached more days of at least 60 min of MVPA with the ACC than given in the PAQ, he/she will be found below the diagonal in one of the vertically lined cells. Participants reaching 60 min MVPA on fewer days than stated in the PAQ will be found in the white cells above the diagonal.

Besides, the difference in days between the two methods was calculated and a t-test was used to detect differences between genders in all age groups. Here the level of significance was set to α = 0.01.

## Results

Reaching the WHO Guidelines of at least 60 min MVPA every day is significantly lower when measured with the ACC than with the PAQ. 9% of the participants met the WHO Guidelines on each day per week when measured with the PAQ, 4% with the ACC. According to the PAQ, more than 60 min MVPA is achieved on a mean_PAQ_ = 3.82 days. When measured with ACC, this goal is achieved on a mean_ACC_ = 2.34 days (*F*_*method*_ = 915.85; *p =* <.001; *f*_*Cohen*_ = .64) (compare Table 3).

**Table 3:** Numbers of days with 60 min of moderate-to vigorous-intensity physical activity (MVPA) as obtained from accelerometer (ACC) measurements and self-reported (PAQ) PA, data are presented as mean and 95%-CI

Group	ACC		PAQ	
	Mean	95%-CI	Mean	95%-CI
Overall	2.42	(2.35–2.50)	3.86	(3.79–3.93)
Boys	2.89	(2.78–3.00)	4.05	(3.95–4.15)
Girls	1.95	(1.85–2.06)	3.67	(3.57–3.76)
6–10 years	3.66	(3.53–3.79)	4.39	(4.26–4.51)
11–13 years	2.08	(1.95–2.22)	3.76	(3.63–3.88)
14–17 years	1.53	(1.40–1.65)	3.43	(3.32–3.54)
Boys				
6–10 years	4.29	(4.10–4.48)	4.54	(4.37–4.72)
11–13 years	2.53	(2.33–2.73)	3.94	(3.76–4.13)
14–17 years	1.85	(1.67–2.03)	3.67	(3.50–3.83)
Girls				
6–10 years	3.03	(2.84–3.22)	4.23	(4.06–4.41)
11–13 years	1.63	(1.45–1.82)	3.57	(3.40–3.74)
14–17 years	1.20	(1.03–1.36)	3.20	(3.05–3.35)

Besides the differences in the detection method, smaller but still large effect sizes can be found between age groups (*F*_*age*_ = 264.2, *p* < .001; *f*_*Cohen*_ = .480). Especially the 6–10-year-olds adhere to the WHO Guidelines significantly more often (16% PAQ, 11% ACC, *p* = .000) on every day of the week compared to 11–13-year-olds (7% PAQ, 2% ACC, *p* < .001) and 14–17-year-olds (4% PAQ, 1% ACC, *p* < .001).

In gender, only medium effect sizes can be found. 11% (PAQ) and 7% (ACC) of boys reached the WHO standard on each day of a week. Girls reach the target on a much lower percentage of days (7% PAQ, 2% ACC, F_gender_ = 134.8, p < .001; *f*_Cohen_ = .25).

**Fig. 2:**
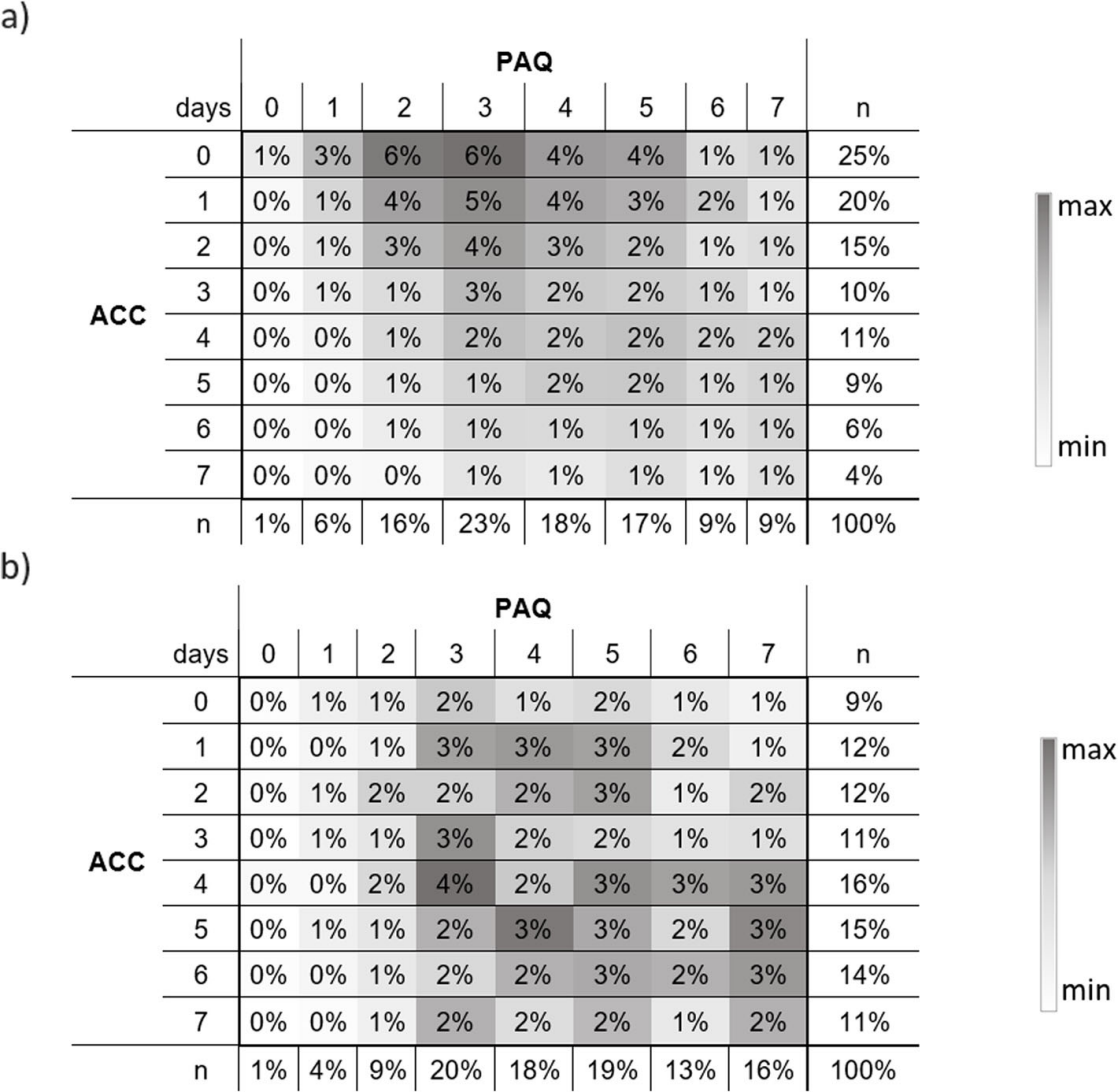
Cross table heat map – numbers of days with MVPA ≥60 min; ACC*PAQ **a**) overall sample **b**) age: 6–10-year-olds; % of participants; heat maps are color-coded (white/min < gray/moderate < black/max)

In the PAQ 68% of all participants stated that they were active on more days than detected with the ACC (upper right corner of the heat map). For 13% of the participants, the answers from the PAQ and the ACC matched, whereas 19% met the Guidelines on more days than stated in the PAQ (see Fig. 2a)). Only the youngest age group shows a different pattern in the heat maps. The remaining groups show patterns similar to that of the overall sample, which is why only these two are shown here. Heat maps for all groups differentiated by age and gender can be found in the supplementary material.

**Fig. 3:**
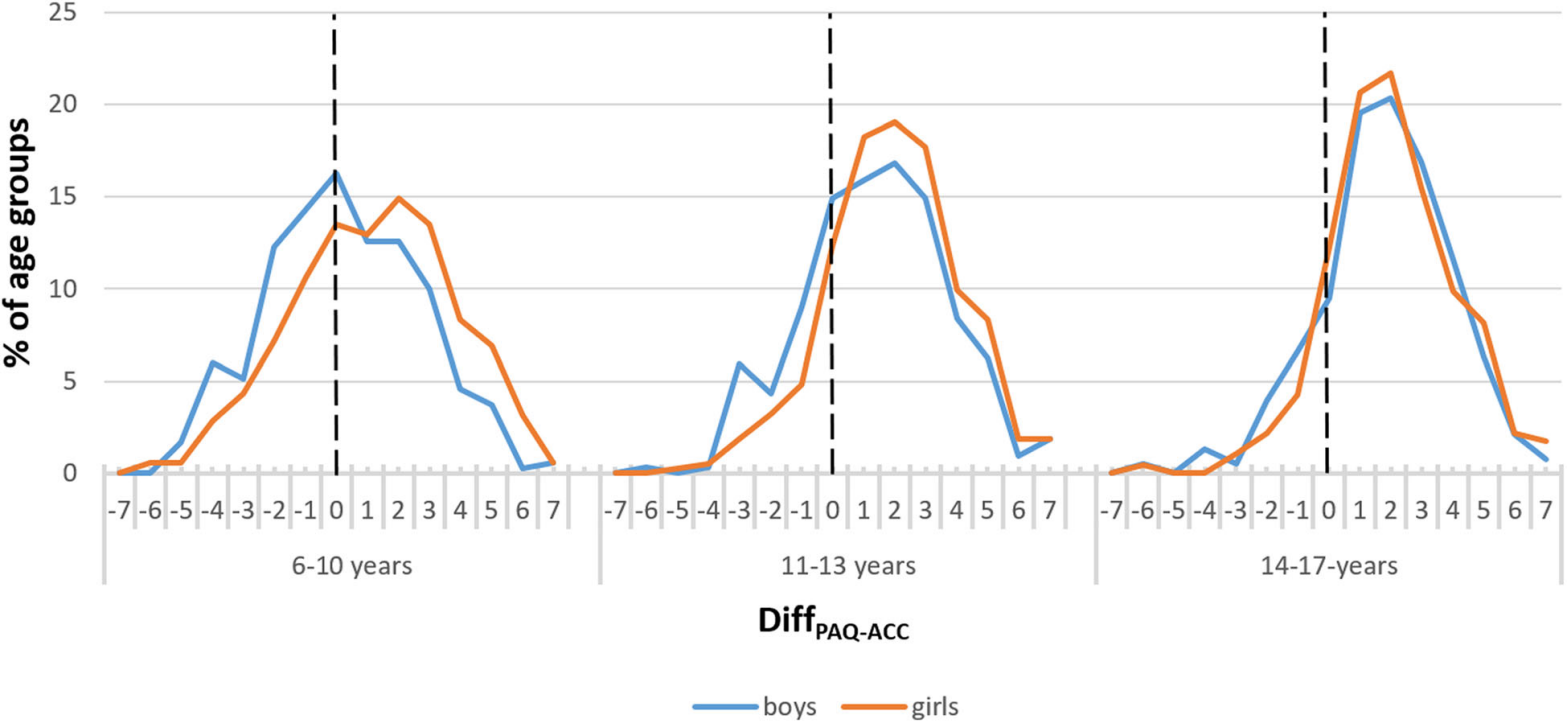
Differences between numbers of days with MVPA ≥60 min measured by PAQ and ACC (Diff_PAQ-ACC_) for the three age groups by gender in %, mean difference for boys and girls, including SD. Zero on the X-axis means the same number of days with MVPA ≥60 min measured by PAQ and ACC

The distribution in the cross table heat maps shows that boys and girls as well as the 11–13-year-olds and the 14–17-year-olds (see supplementary material) did not differ much in distribution from the whole sample. As noted above, girls reached the Guideline on fewer days than boys. Figure 3 shows the differences in the various age groups. The non-overlapping confidence intervals in Table 3 show that gender has a significant influence on the results, but a much bigger influence, and the larger variance can be explained by age.

The 6–10-year-olds differ most from the other groups (see Fig. 2b and supplementary material). 52% of these youngest participants stated more days with 60 min of PA in the PAQ than measured by the ACC. That is 16% less than the average across all participants. Also, the proportion of participants aged 6–10 years with more PA measured by the ACC as stated in the PAQ almost doubled to 33% in contrast to the older participants. The distribution is much smoother with peaks now lying in the estimation corridor (number of days PAQ = number of days ACC), deviations of ± one day, and only a slight overestimation for the PAQ. Figure 3 shows more detailed results for the mean differences of the age groups separated by genders. Here, the difference between boys and girls (6–10 years) in reaching the WHO Guidelines with PAQ and ACC is significant. The youngest boys (meanDiff_boys_ = 0.3d; SD = 2.5) nearly reach their PAQ results with the ACC. The difference in girls (meanDiff_girls_ = 1.2d; SD = 2.6) is almost one day higher in the PAQ than in boys. A significant difference of half a day can be found between genders for 11–13-year-olds (meanDiff_boys_ = 1.4d; meanDiff_girls_ = 2.0d;). No significant difference was found between 14 and 17-year-old boys and girls (meanDiff_boys_ = 1.9d; meanDiff_girls_ = 2.0d).

## Discussion

The study aimed to investigate how children and adolescents in Germany differ in reaching the WHO Guideline of at least 60 min MVPA per day depending on whether PA was self-reported or measured by accelerometer. As expected, the PAQ values were higher than those measured by the ACC but still, both values are alarmingly low.

The low overall adherence to the PA Guideline as obvious from the PAQ can also be found in the results of a recently published pooled data study by the WHO, according to which less than 15% of school-going adolescents aged 11–17 did meet the Guidelines [28]. The lower PA Guideline adherence as measured by the ACC is consistent with the findings of [7, 29, 30] who also reported less light to moderate PA determined by device-based measurements compared to questionnaires. The difference between self-reported and device-based measured PA might be because the PAQ only asks for physically active time, which is subjective and depends on the physical fitness of the participant, as was stated by other studies before [4–6]. Additional, well-known factors that influence the validity of self-reports are recall bias and social desirability [31].

The differences between the results of both methods are significantly smaller for younger children than in the older age groups. Differences between age and gender groups are found in both methods. The older the subjects are, the lower is the proportion of those who meet the WHO Guideline on each day, with girls meeting the guideline less frequently than boys in all age groups. A closer look at the differences between genders in the youngest age group reveals that boys almost match their answers given in the questionnaire with a difference of 0.2 days. The previously mentioned overestimation of PA by the questionnaires [4–6] cannot be found here. The significant difference between the youngest age group and the older ones may be caused by the fact that in this group an external observer (usually a parent) fills out the questionnaire together with the child and may therefore be better able to assess the activity [32]. Found that parent-reported MVPA corresponds to one to two-thirds of the child’s activity measured by the accelerometer. This could be a clue why the gap between the methods is smaller. A more plausible explanation is that the activity patterns of children are more spontaneous, impulsive, and of shorter duration [1, 2]. These short activities, when measured in total, often result in a small amount of light or moderate activity and are poorly captured by questionnaires [33]. Accelerometers, by contrast, register these short and spontaneous movements which are sometimes overlooked when filling out the questionnaire. This does not lead to an overestimation in the questionnaire for small children, but an underestimation due to the short, spontaneous movements not recorded. Since accelerometers measure these movements, this would explain the reduced difference between the two methods.

This is also reflected by the increasing difference between the methods used in the older age groups. The older the participants are, the more structured is their everyday life and the less spontaneous movements are registered by the accelerometer. The familiar overestimation by the questionnaire reoccurs. The older the participants were, the less often they reached the WHO recommendation of 60 min MVPA per day. The lower adherence in older age groups is confirmed by findings of [34, 35] in other European countries and might be due to longer times spent at school or work.

In comparison to boys, girls reach the 60 min MVPA on fewer days in all age groups, but the difference decreases to about half of the initial value with increasing age. The difference in gender and the lower adherence for girls is consistent with the worldwide gender gap of physical activity reported in [28, 35, 36]. Mielke and colleagues [36] found a similar prevalence of inactivity in women and men in a study based on worldwide data of the WHO. Normally, girls and boys might be expected to be equally active until puberty, with the gap starting to open up at this point in time. However, different interests may probably be the reason for this earlier gap - girls tend to be sociable and do esthetic sports, while boys tend to romp, scuffle, and do run-intensive sports. Further examination of the data on PA intensity and sport disciplines in MoMo could give a more detailed answer as to where this difference comes from and where interventions could be useful to close this gap.

### Strength and limitations

The present study is limited to its observational nature and we do not intend to infer causality from paralleled trends or significant correlations. The main goal of MoMo is to track and report PA and fitness of children and adolescents in a nationwide sample, and significant effort was put into collecting representative data from 167 sample points all over the country.

A major strength is the large number of participants and recording of physical activity of each participant by PAQ and ACC. However, this also leads to the restriction that the PAQ assessed PA of an average week, whereas the ACC measured PA during one specific week. An additional comprehensive and elaborate diary was avoided during the week by wearing an ACC. Study participants carried the ACC following the completion of the already very time-consuming fitness test and surveys on activity and health.

Even though the wearing times were very long on average, some participants told us that wearing had been prohibited in some sports competitions like soccer. Wearing electronic devices was forbidden to prevent trainers from having an unfair advantage in knowledge. Even if documented by the non-wear time protocol [14], the unrecorded activities could not be taken into account retrospectively. The manual input of the data from the handwritten non-wear protocol is very time-consuming. Besides, the information in the protocols is very inconsistent and manual input would distort the acceleration data. This missing data could be another link to the difference between PAQ and ACC results in this study. A wearing time of 24 h and a consistent ambulatory assessment for the non-wear time could solve this problem in future studies.

A check of the WHO guidelines is easy to implement with an accelerometer at first glance. However, evaluation results in a multitude of possibilities for implementation. When examining the average time spent with physical activity each week (as now recommended by the new WHO Guidelines of 2020 [37]), days with activity times longer than 60 min would compensate for those with less activity [38]. Still, the daily stimulus is very important in children [39]. This study determined whether the subject was active for at least 60 min or not on each day individually. To look at the exact times spent with MVPA on every single day will result in fewer days of at least 60 min MVPA when both evaluation methods are compared [38]. The main reason, however, since the study was already planned and started in 2014, the questions in the questionnaire still referred to the 2010 WHO Guidelines [10]. Only now the recommendations on youth activity have changed from a recommendation of at least 60 min per day to a recommendation of an average of 60 min per day [37]. This adaptation will require changes in survey questions and sampling methods for future monitoring. However, changing the question wording is unlikely to address the need for PA monitoring among children who, especially at young ages, are unable to answer a complex question about average behavior over the past few days, weeks, or months. In the future, this may require the use of proxy reports from multiple respondents, including parents and teachers, though both may also miss observing large portions of the day [40]. The alternative of asking daily duration for an entire week may be more accurate but increases survey response time. Therefore, measuring daily PA remains a strength of the portable devices for now, and adapting the questionnaires to the new WHO guideline remains a real challenge.

However, we have looked at the accelerometer data with the background of the new WHO guideline. It should be noted, however, that these results cannot be compared with the results of the question used in this study about the number of days on which the subjects have MVPA for more than 60 min. However, if one compares the number of subjects meeting the old versus the new guideline based on the accelerometer data, we see that the percentage increases from 3% to a full 34% of the study participants. This means that 31% of the participants who do not reach 60 min MVPA on all days still have days in the week on which they do so much physical activity that these outweigh the remaining days under the new guideline compared to the old one. This drastically reduces the proportion of children and adolescents who are too inactive, which is also likely to cause some political controversy in the future.

By using an epoch length of one second in MoMo, short activities can be recorded with the accelerometers. This could be another reason why younger children have a more consistent PA outcome with both methods. These short activities are less frequent for older children, which is associated with the fact that an increasing number of older children only practice organized sports. According to MoMo data from previous waves, organized PA in extracurricular activities and sports clubs increased by 8 %, while unorganized PA decreased by 7 % [41].

Apart from PA, many other parameters were collected in MoMo. This results in a multitude of evaluation options, and further examination of the data in MoMo (such as PA intensity, sports disciplines, socioeconomic status, migration status, etc.) will give a more detailed answer as to the reasons behind the differences regarding age and gender.

## Conclusion

Children and adolescents living in Germany and examined within the MoMo and KiGGS studies show a very low adherence to the recommendations given in the 2010 WHO Guideline [10]. These results were confirmed by both survey methods. Surprisingly, the differences in meeting the Guideline between the measurement methods are much smaller for younger children than for older age groups. Future studies should take a deeper look into the underlying cause and verify whether short and spontaneous movements reduce the gap between the methods. Continuous overestimation of the self-report in contrast to the accelerometer was observed in all other age groups. With increasing age, the percentage of compliance with the 2010 WHO Guidelines was found to decreasing, with girls reaching the target with a significantly lower percentage in all age groups. As this study only used PA data, no statements can be made for the underlying cause. The large number of participants that did not reach the WHO Guidelines, however, suggests that PA interventions and further monitoring as well as further analysis of the MoMo data are required. This also is the conclusion drawn by the WHO. It recommends all countries adopt policies and programs to increase the PA of children and adolescents, especially girls [28]. Further examination of the data on PA intensity and sport disciplines in MoMo could give a more detailed answer to the reasons behind the differences regarding age and gender.

Having both self-reported and device-based, measured data will help explain the observed population differences [42]. However, the true value of physical activity probably lies somewhere in between these two methods. The WHO Guideline adaptation in 2020 [37] will require changes in survey questions and sampling methods for future monitoring. To record a more accurate activity profile, a combination of both methods might be a solution. This could be an algorithm to subtract or add the methodological difference if only one method is used. A solution not to lose the activity during non-wear time could be the use of ambulatory assessment in combination with 24 h recording. Triggered e-diaries may ask the subjects for the type of activity performed after certain events have been detected (e.g. device not worn, periods of high activity, or sedentary behavior). Then, non-wear times, their reasons, and activities performed while not wearing the device can be considered uniformly. The participant may be given feedback on how much activity was not recorded and how much was missing to reach the Guidelines. Ambulatory assessment can also be used to ensure that the activity time frames for both methods are consistent by answering the PAQ on the mobile phone at the end of the survey. In this way, the strengths of one method would compensate for the limitations of the other method.

## Supplementary Information

**Additional file 1.**

## Data Availability

Data cannot be shared publicly because of strict ethical requirements with which study investigators are obliged to comply: The Charité/Universitätsmedizin Berlin ethics committee and the Federal Office for the Protection of Data explicitly forbid to make the data publicly available, because the informed consent by study participants did not cover the publication of the data. However, the minimal data set underlying the findings is archived at the Institute of Sports and Sports Science of Karlsruhe Institute of Technology (KIT) and can be accessed on-site by interested researchers. Access requests should be submitted to the Institute of Sports and Sports Science, Karlsruhe Institute of Technology, Engler-Bunte-Ring 15, 76131 Karlsruhe, Germany (info@sport.kit.edu).

## Abbreviations

PA: Physical activity
WHO: World Health Organization
ACC: Accelerometer
PAQ: Physical activity questionnaire
KiGGS: German Health Interview and Examination Survey for Children and Adolescents
RKI: Robert Koch-Institute
MoMo: Motorik-Modul-Study
MVPA: Moderate-to-vigorous-intensity physical activity
