# Supplementary material for: Comparison of self-reported & device-based, measured physical activity among children in Germany
Source: BMC Public Health. 2021 Jun 5;21:1081. doi: 10.1186/s12889-021-11114-y (PMC8180063; doi:10.1186/s12889-021-11114-y)
Supplement: Supplementary file 1 — Additional file 1. [file 12889_2021_11114_MOESM1_ESM.docx]

**Appendices:**

*Cross table Heatmaps – numbers of days with MVPA ≥ 60 min; ACC*PAQ; % of participants; heat maps are color coded (white/min < grey/middle < black/max)*

**Overall:**

| **Crosstable ACC*PAQ - whole sample** | | | | | | | | | | |
| --- | --- | --- | --- | --- | --- | --- | --- | --- | --- | --- |
|  |  | **PAQ** | | | | | | | |  |
|  | days | 0 | 1 | 2 | 3 | 4 | 5 | 6 | 7 | n |
| **ACC** | 0 | 1% | 3% | 6% | 6% | 4% | 4% | 1% | 1% | 25% |
|  | 1 | 0% | 1% | 4% | 5% | 4% | 3% | 2% | 1% | 20% |
|  | 2 | 0% | 1% | 3% | 4% | 3% | 2% | 1% | 1% | 15% |
|  | 3 | 0% | 1% | 1% | 3% | 2% | 2% | 1% | 1% | 10% |
|  | 4 | 0% | 0% | 1% | 2% | 2% | 2% | 2% | 2% | 11% |
|  | 5 | 0% | 0% | 1% | 1% | 2% | 2% | 1% | 1% | 9% |
|  | 6 | 0% | 0% | 1% | 1% | 1% | 1% | 1% | 1% | 6% |
|  | 7 | 0% | 0% | 0% | 1% | 1% | 1% | 1% | 1% | 4% |
|  | n | 1% | 6% | 16% | 23% | 18% | 17% | 9% | 9% | 100% |

| **Crosstable ACC*PAQ - boys** | | | | | | | | | | |
| --- | --- | --- | --- | --- | --- | --- | --- | --- | --- | --- |
|  |  | **PAQ** | | | | | | | |  |
|  | days | 0 | 1 | 2 | 3 | 4 | 5 | 6 | 7 | n |
| **ACC** | 0 | 0% | 2% | 4% | 5% | 3% | 3% | 0% | 1% | 18% |
|  | 1 | 0% | 1% | 3% | 4% | 3% | 4% | 2% | 1% | 17% |
|  | 2 | 0% | 1% | 2% | 4% | 3% | 3% | 1% | 1% | 15% |
|  | 3 | 0% | 0% | 1% | 3% | 2% | 2% | 2% | 1% | 10% |
|  | 4 | 0% | 0% | 1% | 3% | 2% | 3% | 2% | 2% | 13% |
|  | 5 | 0% | 0% | 1% | 2% | 2% | 3% | 1% | 2% | 11% |
|  | 6 | 0% | 0% | 1% | 2% | 2% | 2% | 1% | 2% | 9% |
|  | 7 | 0% | 0% | 0% | 1% | 1% | 2% | 1% | 2% | 7% |
|  | n | 1% | 4% | 14% | 23% | 17% | 20% | 10% | 11% | 100% |

| **Crosstable ACC*PAQ - girls** | | | | | | | | | | |
| --- | --- | --- | --- | --- | --- | --- | --- | --- | --- | --- |
|  |  | **PAQ** | | | | | | | |  |
|  | days | 0 | 1 | 2 | 3 | 4 | 5 | 6 | 7 | n |
| **ACC** | 0 | 1% | 4% | 7% | 8% | 5% | 5% | 2% | 1% | 32% |
|  | 1 | 0% | 2% | 5% | 5% | 4% | 3% | 2% | 1% | 22% |
|  | 2 | 0% | 1% | 4% | 4% | 3% | 2% | 1% | 1% | 15% |
|  | 3 | 0% | 1% | 1% | 2% | 2% | 1% | 1% | 1% | 9% |
|  | 4 | 0% | 0% | 1% | 2% | 2% | 2% | 1% | 1% | 10% |
|  | 5 | 0% | 0% | 1% | 1% | 2% | 1% | 1% | 1% | 7% |
|  | 6 | 0% | 0% | 0% | 1% | 1% | 1% | 1% | 1% | 4% |
|  | 7 | 0% | 0% | 0% | 0% | 0% | 0% | 0% | 0% | 2% |
|  | n | 1% | 9% | 18% | 23% | 19% | 15% | 8% | 7% | 100% |

| **Crosstable ACC*PAQ - 6-10 year olds** | | | | | | | | | | |
| --- | --- | --- | --- | --- | --- | --- | --- | --- | --- | --- |
|  |  | **PAQ** | | | | | | | |  |
|  | days | 0 | 1 | 2 | 3 | 4 | 5 | 6 | 7 | n |
| **ACC** | 0 | 0% | 1% | 1% | 2% | 1% | 2% | 1% | 1% | 9% |
|  | 1 | 0% | 0% | 1% | 3% | 3% | 3% | 2% | 1% | 12% |
|  | 2 | 0% | 1% | 2% | 2% | 2% | 3% | 1% | 2% | 12% |
|  | 3 | 0% | 1% | 1% | 3% | 2% | 2% | 1% | 1% | 11% |
|  | 4 | 0% | 0% | 2% | 4% | 2% | 3% | 3% | 3% | 16% |
|  | 5 | 0% | 1% | 1% | 2% | 3% | 3% | 2% | 3% | 15% |
|  | 6 | 0% | 0% | 1% | 2% | 2% | 3% | 2% | 3% | 14% |
|  | 7 | 0% | 0% | 1% | 2% | 2% | 2% | 1% | 2% | 11% |
|  | n | 1% | 4% | 9% | 20% | 18% | 19% | 13% | 16% | 100% |

| **Crosstable ACC*PAQ - 11-13 year olds** | | | | | | | | | | |
| --- | --- | --- | --- | --- | --- | --- | --- | --- | --- | --- |
|  |  | **PAQ** | | | | | | | |  |
|  | days | 0 | 1 | 2 | 3 | 4 | 5 | 6 | 7 | n |
| **ACC** | 0 | 1% | 2% | 6% | 7% | 5% | 5% | 1% | 2% | 28% |
|  | 1 | 0% | 1% | 4% | 6% | 4% | 3% | 2% | 1% | 21% |
|  | 2 | 0% | 1% | 3% | 5% | 2% | 2% | 1% | 1% | 15% |
|  | 3 | 0% | 1% | 1% | 3% | 2% | 2% | 1% | 1% | 10% |
|  | 4 | 0% | 1% | 1% | 2% | 3% | 3% | 1% | 1% | 12% |
|  | 5 | 0% | 0% | 1% | 1% | 1% | 2% | 1% | 1% | 7% |
|  | 6 | 0% | 0% | 0% | 1% | 1% | 1% | 0% | 1% | 4% |
|  | 7 | 0% | 0% | 0% | 0% | 0% | 0% | 1% | 0% | 2% |
|  | n | 1% | 6% | 15% | 27% | 18% | 18% | 8% | 7% | 100% |

| **Crosstable ACC*PAQ - 14-17 year olds** | | | | | | | | | | |
| --- | --- | --- | --- | --- | --- | --- | --- | --- | --- | --- |
|  |  | **PAQ** | | | | | | | |  |
|  | days | 0 | 1 | 2 | 3 | 4 | 5 | 6 | 7 | n |
| **ACC** | 0 | 1% | 5% | 10% | 9% | 6% | 5% | 1% | 1% | 37% |
|  | 1 | 0% | 2% | 7% | 5% | 4% | 4% | 2% | 1% | 25% |
|  | 2 | 0% | 1% | 4% | 5% | 3% | 2% | 1% | 0% | 16% |
|  | 3 | 0% | 0% | 1% | 2% | 2% | 2% | 1% | 0% | 8% |
|  | 4 | 0% | 0% | 1% | 1% | 1% | 1% | 1% | 1% | 6% |
|  | 5 | 0% | 0% | 0% | 1% | 1% | 1% | 1% | 0% | 5% |
|  | 6 | 0% | 0% | 0% | 0% | 0% | 0% | 0% | 0% | 2% |
|  | 7 | 0% | 0% | 0% | 0% | 0% | 0% | 0% | 0% | 1% |
|  | n | 2% | 8% | 22% | 23% | 18% | 15% | 7% | 4% | 100% |

**Girls:**

| **Crosstable ACC*PAQ - 6-10 year olds** | | | | | | | | | | |
| --- | --- | --- | --- | --- | --- | --- | --- | --- | --- | --- |
|  |  | **PAQ** | | | | | | | |  |
|  | days | 0 | 1 | 2 | 3 | 4 | 5 | 6 | 7 | n |
| **ACC** | 0 | 0% | 1% | 2% | 3% | 3% | 3% | 2% | 1% | 13% |
|  | 1 | 0% | 1% | 1% | 4% | 5% | 3% | 2% | 1% | 18% |
|  | 2 | 0% | 0% | 2% | 2% | 2% | 2% | 1% | 2% | 12% |
|  | 3 | 0% | 1% | 1% | 3% | 2% | 2% | 1% | 1% | 12% |
|  | 4 | 0% | 1% | 1% | 3% | 3% | 3% | 3% | 3% | 16% |
|  | 5 | 0% | 1% | 1% | 3% | 3% | 2% | 2% | 3% | 14% |
|  | 6 | 0% | 0% | 1% | 2% | 1% | 2% | 2% | 2% | 10% |
|  | 7 | 0% | 0% | 0% | 1% | 1% | 1% | 0% | 1% | 5% |
|  | n | 0% | 5% | 10% | 20% | 20% | 19% | 13% | 12% | 100% |

| **Crosstable ACC*PAQ - 11-13 year olds** | | | | | | | | | | |
| --- | --- | --- | --- | --- | --- | --- | --- | --- | --- | --- |
|  |  | **PAQ** | | | | | | | |  |
|  | days | 0 | 1 | 2 | 3 | 4 | 5 | 6 | 7 | n |
| **ACC** | 0 | 1% | 3% | 6% | 10% | 6% | 5% | 2% | 2% | 35% |
|  | 1 | 0% | 2% | 5% | 7% | 3% | 3% | 2% | 0% | 22% |
|  | 2 | 0% | 1% | 3% | 5% | 3% | 2% | 0% | 1% | 16% |
|  | 3 | 0% | 1% | 1% | 3% | 2% | 1% | 1% | 1% | 10% |
|  | 4 | 0% | 1% | 0% | 2% | 3% | 2% | 1% | 2% | 11% |
|  | 5 | 0% | 0% | 1% | 1% | 1% | 1% | 1% | 0% | 4% |
|  | 6 | 0% | 0% | 0% | 1% | 1% | 0% | 0% | 0% | 2% |
|  | 7 | 0% | 0% | 0% | 0% | 0% | 0% | 0% | 0% | 0% |
|  | n | 1% | 9% | 15% | 28% | 18% | 15% | 7% | 6% | 100% |

| **Crosstable ACC*PAQ - 14-17 year olds** | | | | | | | | | | |
| --- | --- | --- | --- | --- | --- | --- | --- | --- | --- | --- |
|  |  | **PAQ** | | | | | | | |  |
|  | days | 0 | 1 | 2 | 3 | 4 | 5 | 6 | 7 | n |
| **ACC** | 0 | 1% | 6% | 12% | 9% | 7% | 6% | 1% | 2% | 44% |
|  | 1 | 0% | 2% | 8% | 5% | 5% | 2% | 2% | 1% | 25% |
|  | 2 | 0% | 1% | 5% | 5% | 3% | 1% | 0% | 0% | 15% |
|  | 3 | 0% | 1% | 1% | 2% | 2% | 1% | 1% | 0% | 7% |
|  | 4 | 0% | 0% | 1% | 1% | 1% | 1% | 1% | 0% | 5% |
|  | 5 | 0% | 0% | 0% | 0% | 1% | 1% | 0% | 0% | 3% |
|  | 6 | 0% | 0% | 0% | 0% | 0% | 0% | 0% | 0% | 1% |
|  | 7 | 0% | 0% | 0% | 0% | 0% | 0% | 0% | 0% | 0% |
|  | n | 2% | 11% | 26% | 21% | 18% | 12% | 6% | 4% | 100% |

**Boys:**

| **Crosstable ACC*PAQ - 6-10 year olds** | | | | | | | | | | |
| --- | --- | --- | --- | --- | --- | --- | --- | --- | --- | --- |
|  |  | **PAQ** | | | | | | | |  |
|  | days | 0 | 1 | 2 | 3 | 4 | 5 | 6 | 7 | n |
| **ACC** | 0 | 0% | 1% | 0% | 1% | 0% | 1% | 0% | 1% | 4% |
|  | 1 | 0% | 0% | 0% | 1% | 1% | 2% | 1% | 0% | 6% |
|  | 2 | 0% | 1% | 1% | 2% | 3% | 3% | 1% | 2% | 13% |
|  | 3 | 0% | 0% | 1% | 3% | 1% | 2% | 1% | 2% | 10% |
|  | 4 | 1% | 0% | 2% | 4% | 1% | 3% | 3% | 3% | 17% |
|  | 5 | 0% | 1% | 1% | 2% | 3% | 3% | 2% | 4% | 16% |
|  | 6 | 0% | 1% | 1% | 2% | 4% | 3% | 3% | 4% | 17% |
|  | 7 | 0% | 0% | 1% | 3% | 2% | 4% | 2% | 4% | 17% |
|  | n | 1% | 3% | 8% | 19% | 16% | 20% | 13% | 19% | 100% |

| **Crosstable ACC*PAQ - 11-13 year olds** | | | | | | | | | | |
| --- | --- | --- | --- | --- | --- | --- | --- | --- | --- | --- |
|  |  | **PAQ** | | | | | | | |  |
|  | days | 0 | 1 | 2 | 3 | 4 | 5 | 6 | 7 | n |
| **ACC** | 0 | 0% | 1% | 5% | 4% | 3% | 4% | 0% | 2% | 20% |
|  | 1 | 1% | 1% | 3% | 5% | 5% | 3% | 2% | 1% | 20% |
|  | 2 | 0% | 0% | 2% | 5% | 2% | 3% | 2% | 0% | 14% |
|  | 3 | 0% | 0% | 1% | 4% | 2% | 2% | 2% | 1% | 11% |
|  | 4 | 0% | 1% | 1% | 3% | 3% | 3% | 1% | 1% | 14% |
|  | 5 | 0% | 0% | 1% | 1% | 1% | 3% | 1% | 1% | 9% |
|  | 6 | 0% | 0% | 0% | 2% | 1% | 2% | 1% | 1% | 7% |
|  | 7 | 0% | 0% | 0% | 0% | 1% | 1% | 2% | 0% | 4% |
|  | n | 1% | 3% | 14% | 25% | 17% | 21% | 10% | 8% | 100% |

| **Crosstable ACC*PAQ - 14-17 year olds** | | | | | | | | | | |
| --- | --- | --- | --- | --- | --- | --- | --- | --- | --- | --- |
|  |  | **PAQ** | | | | | | | |  |
|  | days | 0 | 1 | 2 | 3 | 4 | 5 | 6 | 7 | n |
| **ACC** | 0 | 1% | 3% | 7% | 8% | 5% | 4% | 1% | 1% | 29% |
|  | 1 | 0% | 1% | 5% | 6% | 3% | 5% | 2% | 1% | 25% |
|  | 2 | 1% | 1% | 2% | 5% | 3% | 3% | 1% | 1% | 17% |
|  | 3 | 0% | 0% | 2% | 2% | 2% | 2% | 2% | 0% | 10% |
|  | 4 | 0% | 0% | 1% | 2% | 1% | 2% | 1% | 1% | 8% |
|  | 5 | 0% | 0% | 0% | 2% | 2% | 2% | 1% | 0% | 6% |
|  | 6 | 0% | 0% | 1% | 1% | 0% | 1% | 0% | 1% | 3% |
|  | 7 | 0% | 1% | 0% | 0% | 0% | 0% | 0% | 0% | 1% |
|  | n | 2% | 5% | 18% | 25% | 17% | 19% | 8% | 5% | 100% |
